# Uncertainty: a neglected determinant of health behavior?

**DOI:** 10.3389/fpsyg.2023.1145879

**Published:** 2023-05-12

**Authors:** David Berrigan, David Dean, Nicole Senft Everson, Heather D’Angelo, Patrick Boyd, William M. P. Klein, Paul K. J. Han

**Affiliations:** National Cancer Institute, Division of Cancer Control and Population Sciences, Rockville, MD, United States

**Keywords:** uncertainty, behavior change, trust, ethics, motives

## Abstract

Health behaviors are critical determinants of the well-being of individuals and populations, and understanding the determinants of these behaviors has been a major focus of research. One important determinant that has received little direct attention in past health research is uncertainty: a complex phenomenon that pertains not only to scientific issues regarding the diagnosis, prognosis, prevention, and treatment of health problems, but also to personal issues regarding other important health-related concerns. Here, we make the case for greater attention to uncertainty in health behavior theory and research, and especially to personal uncertainties. We discuss three exemplary types of personal uncertainty—*value* uncertainty, *capacity* uncertainty, and *motive* uncertainty—which relate, respectively, to moral values, capacities to enact or change behaviors, and the motives and intentions of other persons or institutions. We argue that that personal uncertainties such as these influence health behaviors, but their influence has historically been obscured by a focus on other constructs such as self-efficacy and trust. Reconceptualizing and investigating health behavior as a problem of uncertainty can advance both our understanding of the determinants of healthy behaviors and our ability to promote them.

## Introduction

Francis Collins, former director of the NIH, recently called for greater investment in behavioral research (Welland and Kolata, 2021). Dr. Collins was responding to the fact that even though effective and safe COVID-19 vaccines were rapidly developed, extensively tested, and broadly disseminated, public adoption of vaccines and other recommended preventive behaviors was limited, leading to significant avoidable suffering and death. The pandemic has demonstrated the critical importance of understanding not only how to prevent and treat diseases such as COVID-19 but how to promote the human behaviors on which disease prevention and treatment depend. It has showed how problems such as miscommunication, misinformation, and mistrust can prevent people from adopting evidence-based preventive and therapeutic interventions (Cooney, 2022).

Empirical research over the past several decades has been motivated by this goal, and numerous theories have been developed to account for the many factors that influence health behaviors. However, we believe there is one factor that has received conspicuously little direct attention in past health behavior research and theory, despite its profound influence: uncertainty. A cross-cutting human experience, uncertainty has been investigated from many disciplinary perspectives—including communication science, economics, psychology, and sociology—and defined in somewhat different ways across disciplines; however, one useful definition construes uncertainty as a psychological state consisting of the conscious, metacognitive awareness of ignorance about some issue or aspect of reality (Han et al., 2011). A diverse body of research on uncertainty, furthermore, has shown that uncertainty has important psychological effects. These effects can be positive or adaptive, as in experiences of hope, courage, seeking and savoring (Vazard, 2022; Gregory et al., 2023), but more often uncertainty has negative or potentially maladaptive cognitive, emotional, and behavioral effects (Attema et al., 2013; Hillen et al., 2017; Anderson et al., 2019). In the domain of health and healthcare, the COVID-19 pandemic vividly illustrated how uncertainty about a health threat and its management can promote perceptions of vulnerability, mistrust of information, and feelings of fear and worry, as well as negative behavioral consequences such as refusal of recommended risk-reducing interventions and the spread of misinformation and disinformation.

However, uncertainty has received surprisingly little attention in health behavior theory and research. Major theories of health behavior highlight the important causal role of various psychological factors, such as the perceived risk of a health threat or the perceived benefit of risk-reducing action, health-related attitudes, beliefs about social norms, and others. However, these theories largely ignore the role of uncertainty; major health behavior theories seldom, if ever, mention uncertainty explicitly. Yet uncertainty about many different issues can influence health behaviors in important ways. For example, uncertainty about scientific issues such as the diagnosis, prognosis, prevention, or treatment of a health problem can promote pessimistic perceptions of its associated risks or the benefits of risk-reducing actions, and avoidance of decision making—hallmarks of a more general psychological phenomenon known as ambiguity aversion (Ellsberg, 1961). Uncertainty about personal issues such as the moral or existential implications of a health problem may have similar effects, while uncertainty about other personal issues thought to be key determinants of health behavior (e.g., attitudes, beliefs about social norms)—could alter the influence of these factors.

Correspondingly, few published health behavior change interventions (Abraham and Michie, 2008; Michie et al., 2011, 2013) have directly measured or targeted uncertainty. Furthermore, although uncertainty has been acknowledged as a critical focus of efforts to promote informed and shared decision making in medicine and to manage public health crises (Han et al., 2011; Spiegelhalter and Riesch, 2011; Carleton, 2016; Imber, 2017; Han, 2021), the focus has been limited to scientific uncertainties and the provision of information to help resolve them (Han, 2021; Dahm and Crock, 2022). Much less attention has been devoted to personal uncertainties that cannot be resolved through the provision of information alone (Han et al., 2011; Imber, 2017).

The goal of this commentary is to make a case for expanding the focus of health behavior theory and research to include both greater attention to uncertainty in general and especially to personal uncertainty. Focusing on three exemplary personal uncertainties—*value uncertainty*, *capacity uncertainty*, and *motive uncertainty*—we briefly discuss how each influences health behavior but has been historically neglected. These are not the only personal uncertainties that warrant further investigation; however, we show how they represent fruitful starting points for future work. We argue that greater attention to these uncertainties can broaden not only our understanding of the causes of health behaviors, but our capacity to change these behaviors and achieve better health for individuals and populations (Table 1).

**Table 1:** Contrasts between scientific and three types of personal uncertainty related to health.

Uncertainty domain	Uncertainty issue	Related constructs
Scientific	Diagnosis, prognosis, causes, treatment of specific health problems	Accuracy Reliability Validity
Personal: value uncertainty	Moral and ethical values	Value awareness Values clarification
Personal: capacity uncertainty	Capacity to enact or change behavior	Self-efficacy
Personal: motive uncertainty	Intentions and fidelity of others	Medical mistrust

## Value uncertainty

One important personal uncertainty that influences health behaviors pertains to the fundamental human values that guide people’s decisions about these behaviors. This type of uncertainty—which can be termed value uncertainty—affects many health decisions and behaviors, but especially those involving multiple scientifically reasonable options, unfamiliar outcomes, or tradeoffs between different benefits and harms. Adopting a given healthcare intervention or behavior requires people to weigh their personal values and determine which are most important to them. However, people are often uncertain about their own values, and this uncertainty can confound a wide variety of health decisions and behaviors. For example, uncertainty about the values of individual freedom vs. the collective good generates hesitation and controversy about vaccination and other recommended measures for managing COVID-19 and other public health crises (Dupont and Galea, 2022). Personal uncertainties about the value of future parenthood hinders decisions about fertility preservation options for adolescent cancer patients and their parents (Nahata et al., 2021) and choices concerning permanent (e.g., vasectomy) versus temporary measures for birth control among members of the general population. In the realm of disease treatment, personal uncertainty about the relative importance of the value of greater length vs. greater quality of life—and of numerous other desired health states—complicates medical decisions for patients with serious life-limiting illnesses such as cancer. As these examples illustrate, the objects of value uncertainty are diverse and related to cultural, social, and moral norms and beliefs.

Value uncertainty has thus been the implicit focus of calls for greater “values awareness” in end-of-life care (Fischhoff and Barnato, 2019) and more general efforts to promote shared decision making (SDM) in healthcare. An integral component of SDM is “values clarification”: a process of “sorting out what matters to an individual relevant to a given health decision” (Witteman et al., 2016). Explicit and formal value clarification exercises have been incorporated into patient decision aids and shown to promote value-concordant decisions (Witteman et al., 2020) and reduce “decisional conflict”—that is, “personal uncertainty about which course of action to take” (LeBlanc et al., 2009). Although value uncertainty is the core problem driving efforts to promote values awareness and shared decision making, it has seldom been labeled as such or called out explicitly. These efforts have instead focused on value conflicts themselves (e.g., length vs. quality of life, or the expected utility of different health states)—that is, on the *object* as opposed to the *subjective experience* of this uncertainty.

A focus on personal values has brought needed attention to a critical component of medical decision making; however, we believe it has also diverted attention away from other aversive psychological effects of the uncertainty provoked by value conflicts. These aversive psychological effects include negative emotions such as dread, guilt, and regret, and behavioral responses such as information and decision avoidance (Hillen et al., 2017; Anderson et al., 2019). A singular focus on values—as opposed to personal uncertainty about these values—has thus left unaddressed the task of helping people cope with these negative psychological effects and to accept and live with their uncertainty. This is just as important a task as values clarification given that conflicts between many personal values are fundamentally irresolvable, no matter how well-clarified they may be. People facing difficult health decisions are often left with some level of value uncertainty that must somehow be palliated, not cured. To our knowledge, however, the palliation of value uncertainty has not been a focus of efforts to promote value awareness and shared decision making in healthcare. Addressing this gap requires expanding the focus of research and practice from values *per se* to people’s experience of uncertainty about these values.

## Capacity uncertainty

A second important personal uncertainty pertains to individuals’ capacity to engage in health behaviors. This type of uncertainty has been partially captured through a large body of health behavior theory and research on the construct of self-efficacy. As originally conceptualized by Bandura, self-efficacy represents “People’s judgments of their capabilities to organize and execute courses of action required to attain designated types of performances^”^(Bandura, 1986). Subjective self-efficacy assessments determine whether any given behavior “will be initiated, how much effort will be expended, and how long it will be sustained in the face of obstacles and aversive experiences”(Bandura, 1977). Self-efficacy can be directed toward a variety of capacities and goals, and empirical research supports its causal role in the initiation and maintenance of various health-promoting behaviors. It is also among the most influential predictors of health behavior in many studies of health behavior theory (Williams and Rhodes, 2016).

Yet as important as research on this construct has been, it has disregarded the important dimension of uncertainty in people’s judgments of their behavioral capacities. Regardless of how individuals might judge their capabilities to organize and execute a given course of action, they may be uncertain about these judgments; they may not be sure about their ability to behave in a particular way or achieve some specified goal or what exact rating they should provide on a self-efficacy measure. As the literature on pluralistic ignorance suggests, people are often uncertain about their actual abilities to adopt behaviors contrary to perceived social norms (Prentice and Miller, 1996). This distinct, second-order uncertainty—pertaining to self-efficacy assessments themselves—may alter their effects on health behaviors. However, this higher-order uncertainty is not captured in health behavior theories or existing measures of self-efficacy—which instead assume certainty in people’s assessments of their capacity to engage in specific behaviors.

We believe this assumption is questionable and that health behavior theory and research should be expanded to capture people’s uncertainty about their self-efficacy. A broader focus on uncertainty could help account for limitations in the explanatory power of self-efficacy, which might otherwise be attributed to measurement error or other factors. Such an expanded focus, however, requires reliable and valid methods of measuring uncertainty about self-efficacy—which might include novel measures or new analytic approaches to “do not know” responses on existing self-efficacy measures. The latter approach has been advocated for in studies of risk perceptions—another important behavioral determinant for which second-order uncertainty (about risk perceptions themselves) can provoke distinct behavioral responses (Han et al., 2006; Hay et al., 2021). A similar focus on people’s uncertainty about their self-efficacy directs attention to the question of what causal factors and pathways determine the level of this uncertainty and its psychological effects. Research to answer this question can expand not only our understanding of health behavior but our ability to change these behaviors.

## Motive uncertainty

A third important type of personal uncertainty, and one with direct relevance to health equity as well as health decisions and behaviors, pertains to the motives—that is, the underlying intentions and fidelity—of other persons and institutions including health experts. For example, there may be uncertainty about whether physicians’ recommendations are motivated by financial incentives or whether a given healthcare provider treats all patients equally, regardless of demographic factors such as age and race. This type of uncertainty has been the implicit focus of research on several phenomena. Principal among these is medical mistrust, a phenomenon captured by various related constructs, including mistrust and distrust in medicine (Hall et al., 2001, 2002), healthcare, healthcare providers, the medical system, and medical science (Siegrist and Zingg, 2014; Jaiswal and Halkitis, 2019; Baker, 2020; Breakwell, 2020). Medical mistrust has been construed in several ways (Govier, 1994; Armstrong et al., 2008), but one general definition is “the belief that the entity that is the object of mistrust is acting against one’s best interest or well-being” (Jaiswal and Halkitis, 2019). Empirical research suggests that mistrust in health care providers and institutions (Jaiswal and Halkitis, 2019) is an important cause of various negative outcomes, including adverse health states, patient experiences with care, and avoidance of healthcare, whereas trust is an important cause of various positive outcomes (Birkhäuer et al., 2017).

Mistrust is arguably not the root cause of these outcomes but an intervening variable—a negative psychological response to a more fundamental factor: uncertainty about the motives of others. *Medical* mistrust represents a specific negative response to uncertainty about the motives of medical providers or experts—a pessimistic appraisal of the fidelity of medical professionals, institutions, or systems to an individual’s personal interests (Hall et al., 2001, 2002). But mistrust is not the only psychological response to motive uncertainty; trust or faith in medical experts are also possible. Which of these responses predominates in any medical situation depends on numerous factors including individuals’ other responses to motive uncertainty (e.g., information seeking, relationship building), as well as the nature and quality of their past and current interactions with medical experts.

Conceptualizing medical mistrust as not simply a cause of health behaviors but an effect of motive uncertainty can expand our understanding by directing attention upstream, to the causal antecedents and pathways that produce motive uncertainty itself, and that lead from uncertainty to mistrust. More research is needed to answer the critical question of not only how trust and mistrust influence adoption and maintenance of health-related behaviors, but how and why motive uncertainty arises and produces mistrust in the first place, as opposed to other potential outcomes. Various factors—including systemic racism, personal and vicarious life experiences, intersubjective norms, empathic communication, and others—may increase or decrease motive uncertainty and moderate or mediate its effects on medical mistrust or trust. We believe that shifting the focus from mistrust to the motive uncertainty that causally precedes it may ultimately promote the development of interventions that meaningfully address sources of medical mistrust and enhance the adoption of healthier behaviors by individuals and populations.

## Discussion

Uncertainty is a critical but often neglected determinant of health behaviors and decisions, and we have argued that three types of personal uncertainty in particular—value, capacity, and motive uncertainty—have received insufficient attention in past health behavior research and theory. We believe that solving this problem—acknowledging uncertainty as a fundamental determinant of health behavior—has potential benefits for health care research and practice, but realizing these benefits will require additional research to address three broad needs.

The first need is to develop reliable and valid measures of the uncertainties experienced by patients, members of the public, and other key stakeholders. Ideally, such measures should also assess different types of uncertainty, including not only scientific uncertainty, but various personal uncertainties including value, self-efficacy, motive uncertainty, and many others. Development of such measures will also require further work exploring the nature of ‘uncertainty’ in different settings such as those discussed here. Additional research is also needed to improve the measurement of individual differences in “uncertainty tolerance”—an important construct signifying the set of individuals’ negative and positive psychological responses to uncertainty—which may moderate the effects of different uncertainties on health behavior (Hillen et al., 2017).

The second important need, which builds upon the first, is to expand empirical research to elucidate the causal factors and pathways that link uncertainty and health behaviors, and to expand existing health behavior theories to explicitly account for uncertainty. More research is needed to elucidate why and how various uncertainties arise and what factors both moderate and mediate their effects on health behaviors. The ultimate need is to clarify how uncertainty relates to other key constructs in theories of health behavior, and to integrate uncertainty within these theories. At a minimum, health behavior theories may need to acknowledge the potential role of uncertainty more explicitly as a moderator the influence of various established factors—such as self-efficacy, perceived risk, and many others. But health behavior theories may also need to be revised and expanded to include uncertainty alongside these other factors.

The third and most important need for both clinical and public health practice is to develop new interventions to address uncertainties that impede people’s adoption of healthy behaviors and thereby promote well-being. We have focused on value, capacity, and motive uncertainties, but many other personal uncertainties also influence health behaviors and represent important targets for behavior change interventions. Different uncertainties may require different interventions; for example, addressing value uncertainty may require interventions to help people to tolerate conflicting values, while addressing motive uncertainty may require interventions that can help healthcare professionals to engage more effectively with communities to build trusting relationships (Kraft et al., 2018). However, a common element of interventions that target uncertainty to promote behavior change is a fundamental shift in the focus and goals of intervention: from informational to non-informational strategies aimed not at convincing people to adopt a behavior, but at working with them to manage their uncertainties about these behaviors. When possible, uncertainties that discourage healthy behaviors should be reduced. However, many personal uncertainties—e.g., about which values ought to guide a particular decision, how capable one is of successfully enacting a given healthy behavior, or whether a health expert making a recommendation is acting in one’s best interests—cannot be reduced with more information. Promoting behavior change requires emotional and relational support aimed at palliating rather than curing these uncertainties—that is, helping people accept and cope with them. More research is needed to identify the most effective strategies to achieve these goals.

Importantly, making uncertainty a more central focus of health behavior research, theory, practice, or policy does not require replacing existing health behavior theories or constructs such as values, self-efficacy, or medical mistrust, nor does it require abandoning strategies that are based upon them. It requires expanding these efforts by integrating the construct of uncertainty more explicitly and directly within them—that is, treating health behavior change and relationships with health care systems as a problem, in part, of uncertainty. Establishing the value of such an expanded focus will require more research; however, we believe it will ultimately enhance both our understanding of health behaviors and our ability to intervene to promote behaviors that improve the health of individuals and populations.

## Data availability statement

The original contributions presented in the study are included in the article/supplementary material, further inquiries can be directed to the corresponding author/s.

## Author contributions

DB, DD, NS, HD'A, PB, WK, and PH contributed to conceiving these ideas and contributed to reviewing and editing the resulting paper. DB and PH drafted the paper. All authors contributed to the article and approved the submitted version.

## Conflict of interest

The authors declare that the research was conducted in the absence of any commercial or financial relationships that could be construed as a potential conflict of interest.

## Publisher’s note

All claims expressed in this article are solely those of the authors and do not necessarily represent those of their affiliated organizations, or those of the publisher, the editors and the reviewers. Any product that may be evaluated in this article, or claim that may be made by its manufacturer, is not guaranteed or endorsed by the publisher.

## References

[ref1] Abraham C. Michie S. (2008). A taxonomy of behavior change techniques used in interventions. Health Psychol. 27, 379–387. doi: 10.1037/0278-6133.27.3.379 18624603

[ref2] Anderson E. C. Carleton R. N. Diefenbach M. Han P. K. J. (2019). The relationship between uncertainty and affect. Front. Psychol. 10:2504. doi: 10.3389/fpsyg.2019.02504, PMID: 31781003 PMC6861361

[ref3] Armstrong K. McMurphy S. Dean L. T. Micco E. Putt M. Halbert C. H.. (2008). Differences in the patterns of health care system distrust between blacks and whites. J. Gen. Intern. Med. 23, 827–833. doi: 10.1007/s11606-008-0561-9, PMID: 18299939 PMC2517897

[ref4] Attema A. E. Brouwer W. B. I'Haridon O. (2013). Prospect theory in the health domain: a quantitative assessment. J. Health Econ. 32, 1057–1065. doi: 10.1016/j.jhealeco.2013.08.006, PMID: 24103499

[ref5] Baker D. W. (2020). Trust in health care in the time of COVID-19. JAMA 324, 2373–2375. doi: 10.1001/jama.2020.23343 33320208

[ref6] Bandura A. (1977). Self-efficacy: toward a unifying theory of behavioral change. Psychol. Rev. 84, 191–215. doi: 10.1037//0033-295x.84.2.191, PMID: 847061

[ref7] Bandura A. (1986). Social Foundations of thought and Action: A Social Cognitive Theory. Englewood Cliffs: Prentice Hall.

[ref8] Birkhäuer J. Gaab J. Kossowsky J. Hasler S. Krummenacher P. Werner C.. (2017). Trust in the health care professional and health outcome: a meta-analysis. PLoS One 12:e0170988. doi: 10.1371/journal.pone.0170988, PMID: 28170443 PMC5295692

[ref9] Breakwell G. M. (2020). Mistrust, uncertainty and health risks. Contemp Soc Sci 15, 504–516. doi: 10.1080/21582041.2020.1804070

[ref10] Carleton R. N. (2016). Into the unknown: a review and synthesis of contemporary models involving uncertainty. J. Anxiety Disord. 39, 30–43. doi: 10.1016/j.janxdis.2016.02.007, PMID: 26945765

[ref11] Cooney E. (2022). ‘I’m Deeply Concerned’: Francis Collins on Trust in Science, How Covid Communications Failed, and His Current Obsession. Stat; Available at: https://www.statnews.com/2022/09/19/francis-collins-trust-science-covid-communication-failures/ (Accessed January 5, 2023).

[ref12] Dahm M. R. Crock C. (2022). Understanding and communicating uncertainty in achieving diagnostic excellence. JAMA 327, 1127–1128. doi: 10.1001/jama.2022.2141, PMID: 35238876

[ref13] Dupont S. C. Galea S. (2022). Science, competing values, and trade-offs in public health – the example of Covid-19 and masking. N. Engl. J. Med. 387, 865–867. doi: 10.1056/NEJMp2207670, PMID: 36053231

[ref14] Ellsberg D. (1961). Risk, ambiguity, and the savage axioms. Q. J. Econ. 75, 643–669. doi: 10.2307/1884324

[ref15] Fischhoff B. Barnato A. E. (2019). Value awareness: a new goal for end-of-life decision making. MDM Policy Pract 4:2381468318817523. doi: 10.1177/2381468318817523, PMID: 30746498 PMC6360469

[ref16] Govier T. (1994). Is it a jungle out there? Trust, distrust and the construction of social reality. J Dialogue 33, 237–252. doi: 10.1017/S0012217300010519

[ref17] Gregory A. L. Quoidbach J. Haase C. M. Piff P. K. (2023). Be here now: perceptions of uncertainty enhance savoring. Emotion 23, 30–40. doi: 10.1037/emo0000961 34323525

[ref18] Hall M. A. Dugan E. Zheng B. Mishra A. K. (2001). Trust in physicians and medical institutions: what is it, can it be measured, and does it matter? Milbank Q. 79, 613–639. doi: 10.1111/1468-0009.00223, PMID: 11789119 PMC2751209

[ref19] Hall M. A. Zheng B. Dugan E. Camacho F. Kidd K. E. Mishra A.. (2002). Measuring patients' trust in their primary care providers. Med. Care Res. Rev. 59, 293–318. doi: 10.1177/1077558702059003004 12205830

[ref20] Han P.K.J. (2021). Uncertainty in Medicine: A Framework for Tolerance. New York: Oxford University Press.

[ref21] Han P. K. Coates R. J. Uhler R. J. Breen N. (2006). Decision making in prostate-specific antigen screening National Health Interview Survey, 2000. Am. J. Prev. Med. 30, 394–404. doi: 10.1016/j.amepre.2005.12.006 16627127

[ref22] Han P. K. Klein W. M. Arora N. K. (2011). Varieties of uncertainty in health care: a conceptual taxonomy. Med. Decis. Mak. 31, 828–838. doi: 10.1177/0272989x11393976 PMC3146626 22067431

[ref23] Hay J. L. Schofield E. Kiviniemi M. Waters E. A. Chen X. Kaphingst K.. (2021). Examining strategies for addressing high levels of 'I don't know' responding to risk perception questions for colorectal cancer and diabetes: an experimental investigation. Psychol. Health 36, 862–878. doi: 10.1080/08870446.2020.1788714, PMID: 32876479 PMC7952023

[ref24] Hillen M. A. Gutheil C. M. Strout T. D. Smets E. M. A. Han P. K. J. (2017). Tolerance of uncertainty: conceptual analysis, integrative model, and implications for healthcare. Soc. Sci. Med. 180, 62–75. doi: 10.1016/j.socscimed.2017.03.024, PMID: 28324792

[ref25] Imber J. B. (2017). How navigating uncertainty motivates Trust in Medicine. AMA J. Ethics 19, 391–398. doi: 10.1001/journalofethics.2017.19.4.mhst1-1704, PMID: 28430574

[ref26] Jaiswal J. Halkitis P. N. (2019). Towards a more inclusive and dynamic understanding of medical mistrust informed by science. Behav. Med. 45, 79–85. doi: 10.1080/08964289.2019.1619511, PMID: 31343962 PMC7808310

[ref27] Kraft S. A. Cho M. K. Gillespie K. Halley M. Varsava N. Ormond K. E.. (2018). Beyond consent: building trusting relationships with diverse populations in precision medicine research. Am. J. Bioeth. 18, 3–20. doi: 10.1080/15265161.2018.1431322, PMID: 29621457 PMC6173191

[ref28] LeBlanc A. Kenny D. A. O'Connor A. M. Légaré F. (2009). Decisional conflict in patients and their physicians: a dyadic approach to shared decision making. Med. Decis. Mak. 29, 61–68. doi: 10.1177/0272989x08327067, PMID: 19196706

[ref29] Michie S. Richardson M. Johnston M. Abraham C. Francis J. Hardeman W.. (2013). The behavior change technique taxonomy (v1) of 93 hierarchically clustered techniques: building an international consensus for the reporting of behavior change interventions. Ann. Behav. Med. 46, 81–95. doi: 10.1007/s12160-013-9486-6, PMID: 23512568

[ref30] Michie S. van Stralen M. M. West R. (2011). The behaviour change wheel: a new method for characterising and designing behaviour change interventions. Implement. Sci. 6:42. doi: 10.1186/1748-5908-6-42, PMID: 21513547 PMC3096582

[ref31] Nahata L. Olsavsky A. Dattilo T. M. Lipak K. G. Whiteside S. Yeager N. D.. (2021). Parent-adolescent concordance regarding fertility perspectives and sperm banking attempts in adolescent males with cancer. J. Pediatr. Psychol. 46, 1149–1158. doi: 10.1093/jpepsy/jsab069, PMID: 34333651 PMC8561244

[ref32] Prentice D. A. Miller D. T. (1996). Pluralistic ignorance and the perpetuation of social norms by unwitting actors. Adv. Exp. Soc. Psychol. 28, 161–209. doi: 10.1016/S0065-2601(08)60238-5

[ref33] Siegrist M. Zingg A. (2014). The role of public trust during pandemics: implications for crisis communication. Eur. Psychol. 19, 23–32. doi: 10.1027/1016-9040/a000169

[ref34] Spiegelhalter D. J. Riesch H. (2011). Don't know, can't know: embracing deeper uncertainties when analysing risks. Philos Trans R Soc A 369, 4730–4750. doi: 10.1098/rsta.2011.0163, PMID: 22042895

[ref35] Vazard J. (2022). Feeling the unknown: emotions of uncertainty and their valence. Erkenntnis. doi: 10.1007/s10670-022-00583-1

[ref36] Welland N. Kolata G. (2021). Francis Collins, Who Guided N.I.H. through Covid-19 Crisis, Is Exiting. New York Times October 5, 2021.

[ref37] Williams D. M. Rhodes R. E. (2016). The confounded self-efficacy construct: conceptual analysis and recommendations for future research. Health Psychol. Rev. 10, 113–128. doi: 10.1080/17437199.2014.941998, PMID: 25117692 PMC4326627

[ref38] Witteman H. O. Julien A. S. Ndjaboue R. Exe N. L. Kahn V. C. Angie Fagerlin A.. (2020). What helps people make values-congruent medical decisions? Eleven strategies tested across 6 studies. Med. Decis. Mak. 40, 266–278. doi: 10.1177/0272989x20904955, PMID: 32428426

[ref39] Witteman H. O. Scherer L. D. Gavaruzzi T. Pieterse A. H. Fuhrel-Forbis A. Chipenda Dansokho S.. (2016). Design features of explicit values clarification methods: a systematic review. Med. Decis. Mak. 36, 453–471. doi: 10.1177/0272989x15626397, PMID: 26826032

